# Comparative study of the effect of rivaroxaban and fondaparinux on monocyte’s coagulant activity and cytokine release

**DOI:** 10.1186/2162-3619-3-30

**Published:** 2014-12-17

**Authors:** Marc Laurent, Ulrich Joimel, Rémi Varin, Lionel Cazin, Caroline Gest, Veronique Le-Cam-Duchez, Jian Jin, Jielin Liu, Jean-Pierre Vannier, He Lu, Jeannette Soria, Hong Li, Claudine Soria

**Affiliations:** Laboratory MERCI (EA 3829), Faculty of Medicine and Pharmacy, CHU Rouen, Rouen, France; INSERM UMR 965, Lariboisiere Hospital, University of Paris Diderot, Paris, France; INSERM UMR_S1165, IUH, University of Paris Diderot, Saint Louis Hospital, Paris, France; School of Pharmaceutical Sciences, Jiangnan University, Wuxi, Jiangsu 214122 China; Research center of Tissue engineering and stem cells, Guiyang Medical University, 550004 Guiyang, China

**Keywords:** Coagulant, Fondaparinux, Rivaroxaban, Monocyte, Cytokine

## Abstract

**Objectives:**

Tissue factor (TF) exposed on activated monocytes and macrophages is involved in thrombosis through activation of factor X and cytokine release, responsible for inflammation and thrombosis. We investigated the effect of two anti-factor Xa drugs: rivaroxaban, a direct anti-Xa inhibitor, and fondaparinux, an antithrombin dependent anti-Xa inhibitor, on monocyte/macrophage procoagulant activity and cytokine release.

**Methods:**

Rivaroxaban and fondaparinux were tested at pharmacological concentrations on LPS-activated monocytes and on THP-1 cells, a human monocytic cell line, to assess 1) TF expression by flow cytometry 2) prothrombinase activity by its coagulant activity and 3) cytokine release in cell supernatants by antibody based cytokine array and ELISA for IL-8 and TNFα.

**Results and conclusion:**

Rivaroxaban and fondaparinux did not modify TF expression level on activated cells. In contrast procoagulant activity associated to monocytes and macrophages was dose dependently inhibited by rivaroxaban, but not significantly by fondaparinux. These results could explain why patients undergoing major orthopedic surgery with rivaroxaban prophylaxis were able to achieve significant reductions in venous thromboembolism, compared with drugs commonly used, i.e. fondaparinux and low molecular weight heparin. In addition, rivaroxaban and fondaparinux suppressed some chemokine secretion produced by activated macrophages. This may also contribute to their antithrombotic effect in clinic.

## Background

Newer antithrombotic drugs have been developed to improve efficacy and safety for prevention and treatment of venous and arterial thrombosis. Drug candidates are designed to achieve a direct specific inhibition of coagulation factors that are presumed to play an important role in thrombogenesis. Among them, there are new orally active antithrombotic agents targeting thrombin or factor Xa that have previously been approved in therapeutics [1].

Fondaparinux (Arixtra®) is a synthetic penta saccharide that selectively binds to antithrombin III, potentiating factor Xa neutralization thus inhibiting thrombin formation [2, 3]. Rivaroxaban (Xarelto®) is a direct factor Xa inhibitor which binds directly to the Xa active site, blocking its activity [4]. Rivaroxaban is 100,000-fold more selective for FXa than for other biological proteases such as thrombin, plasmin, factor VIIa or factor IXa. Both, fondaparinux and rivaroxaban inhibit thrombin generation in plasma when the coagulation cascade is triggered by tissue factor (TF).

Activated coagulation factor X (FXa) is a major target for designing anticoagulant drugs: it is located at the convergence of the intrinsic and extrinsic coagulation cascade, and activation of one molecule of factor X results in the generation of 1000 molecules of thrombin [5, 6]. Factor Xa assembled along with factor Va on the cell surface results in prothrombinase complex formation, a potent activator of prothrombin. This complex enhances factor Xa catalytic efficacy by several orders of magnitude in the rate of factor II activation into thrombin [7].

It is well established that in thrombosis, FXa generation results from an activation of coagulation cascade initiated by TF expressed on activated endothelial cells and activated monocytes/macrophages [8], while under physiological conditions, endothelial cells and monocytes /macrophages do not express TF and TF activity is counterbalanced by TFPIs [9]. Endothelial cells secrete TFPIα and express TFPIβ on the cell surface and TFPIs was found bound to the surfaces of monocytes [10]. TFPIα and TFPIβ inhibit both TF-factor VIIa-dependent factor Xa (FXa) generation and free FXa. TFPIα inhibits prothrombinases in the presence of Protein S and factor Va [9, 10]. In some pathological conditions, TF is over-expressed by a variety of cells, including monocytes and tumor cells, and the correct balance between TF and TFPI is disrupted [9, 11, 12]. Monocytes and macrophages are the major cell type developing pro-coagulant activity through TF expression induced by inflammatory stimuli [13–17]. Monocyte thrombogenic activity could contribute to thrombotic risk in surgery [18] and cancer [19, 20]. In fact, tumor associated macrophages express a significantly higher level of TF than control cells [21].

Clinical studies have confirmed the major contribution of TF expressed on activated monocytes in thrombosis [22–24]. After plaque injury, as observed in angioplasty, exposure of cellular and extracellular TF to circulating blood play a pivotal role in mediating fibrin-rich thrombus formation leading to acute coronary syndromes [25]. TF expressed on monocytes/macrophages is up-regulated by inflammatory cytokines and oxidized lipids from the plaque [26]. Moreover, activated macrophages also acquire other functional properties, including cytokine production that may significantly participate in autocrine and paracrine signaling among leukocytes and vascular endothelial cells [27, 28].

In addition to the extrinsic activation mediated by tissue factor-factor VIIa, after inflammatory stimuli, monocytes can initiate coagulation in an alternative procoagulant response due to the binding of the zymogen factor X to the integrin Mac-1 (CD11b/CD18) triggering monocyte degranulation and cathepsin G activation of factor X, which catalyzes the cleavage of FX at a novel Leu177- Leu178 peptide bond to form an active protease [21, 28–31]. The newly generated factor Xa remains associated with the monocyte membrane, and promotes procoagulant activity and thrombin formation [9, 21, 28–31].

Finally, several lines of evidence suggest that both FXa and thrombin, by binding to protease-activated receptors (PAR) expressed on monocytes also elicit a cellular response inducing inflammatory cytokine release [32]. PAR-1 expressed by monocytes is cleaved by thrombin and triggered thrombin-dependent inflammation [33]. PAR-2, expressed on monocytes and macrophages is cleaved by FXa [34], contributing to a chronic inflammatory state [28, 29].

Since FXa acts as the key molecule in coagulation amplification (one molecule of factor Xa generating approximately 1000 molecules of thrombin) [5, 6], and that activated monocytes/macrophages participate in thrombosis, inflammation and atherosclerotic complications, we compared the effects of the direct FXa inhibitor rivaroxaban and the indirect imhibitor fondaparinux on both coagulation activation and chemokine secretion. Rivaroxaban is a small chemical molecule and a specific inhibitor of factor Xa. In contrast, fondaparinux is a synthetic molecule composed of five saccharides. It binds to antithrombin and activates antithrombin. Once antithrombin forms a complex with fondaparinux, it selectively neutralizes the activity of factor Xa. Both monocytes isolated from healthy volunteers and human THP-1 macrophages were included in this study.

## Results

### Increase in TF expressed on stimulated monocyte surface

**Figure 1:**
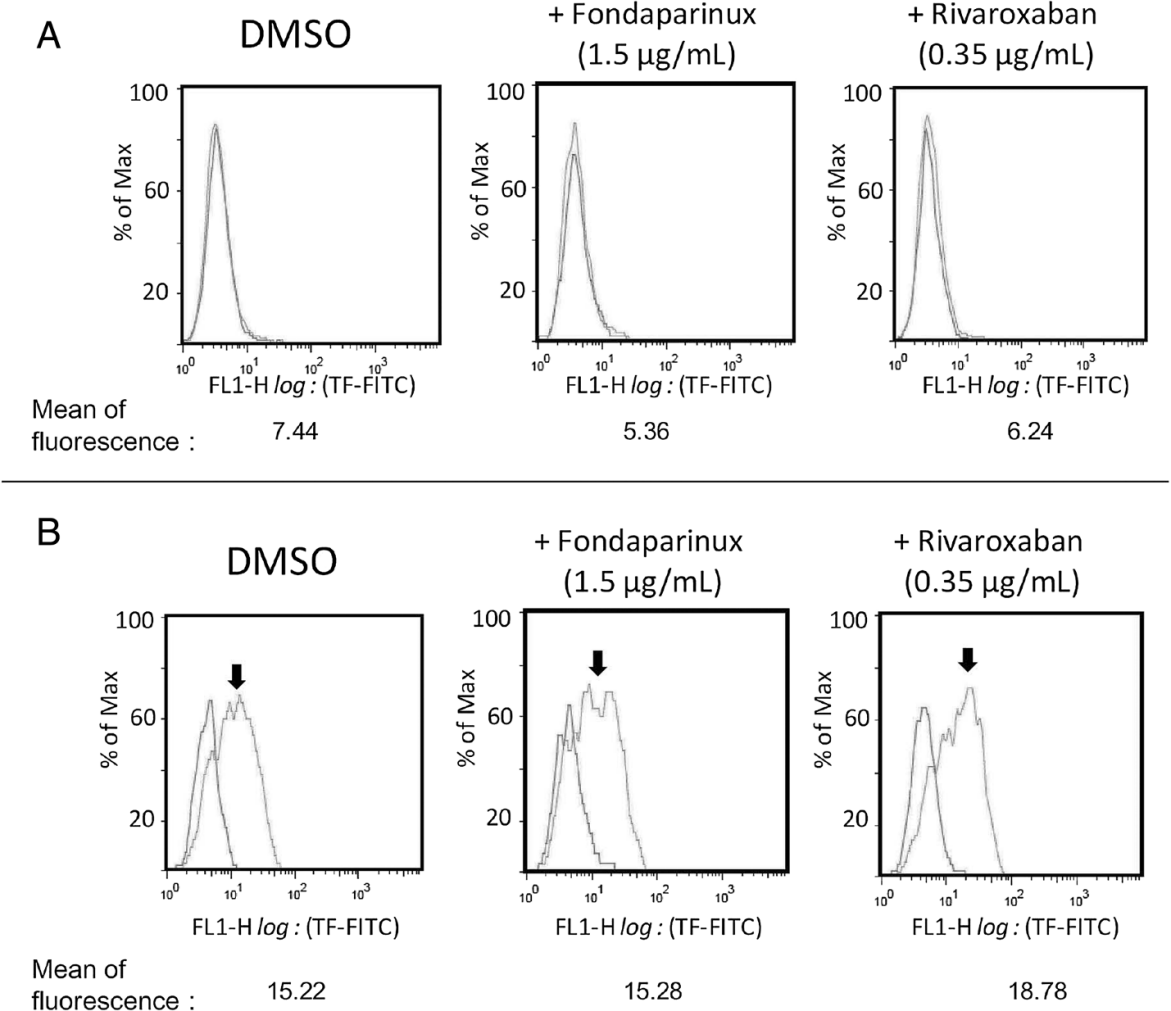
**Flow cytometry analysis of TF expression on non-activated monocytes and LPS-activated monocytes.** The monocytes either unstimulated **(A)** or LPS-stimulated **(B)** were incubated in presence of buffer (control) or fondaparinux (1.5 μg/ml) or rivaroxaban (0.35 g/ml) for 24 hours. Then the cells were washed and incubated with anti-TF antibodies and analyzed in the flow cytometry. The arrows showed increased TF expression in the LPS-activated monocytes. The means of fluorescence intensity (MFI) of each sample minus MFI obtained from the controls were shown.

In unstimulated cells, the TF expression was very low with the mean of fluorescence close to the control without labeled specific anti-TF antibodies as shown in Figure 1A. An important increase in TF expression was observed in LPS-activated monocytes (Figure 1B). The TF expression level in the LPS-activated monocytes was not interfered by the addition in culture media of rivaroxaban or fondaparinux.

### Effect of rivaroxaban and fondaparinux on the endogenous procoagulant activity on monocytes and macrophages surface in plasma

**Figure 2:**
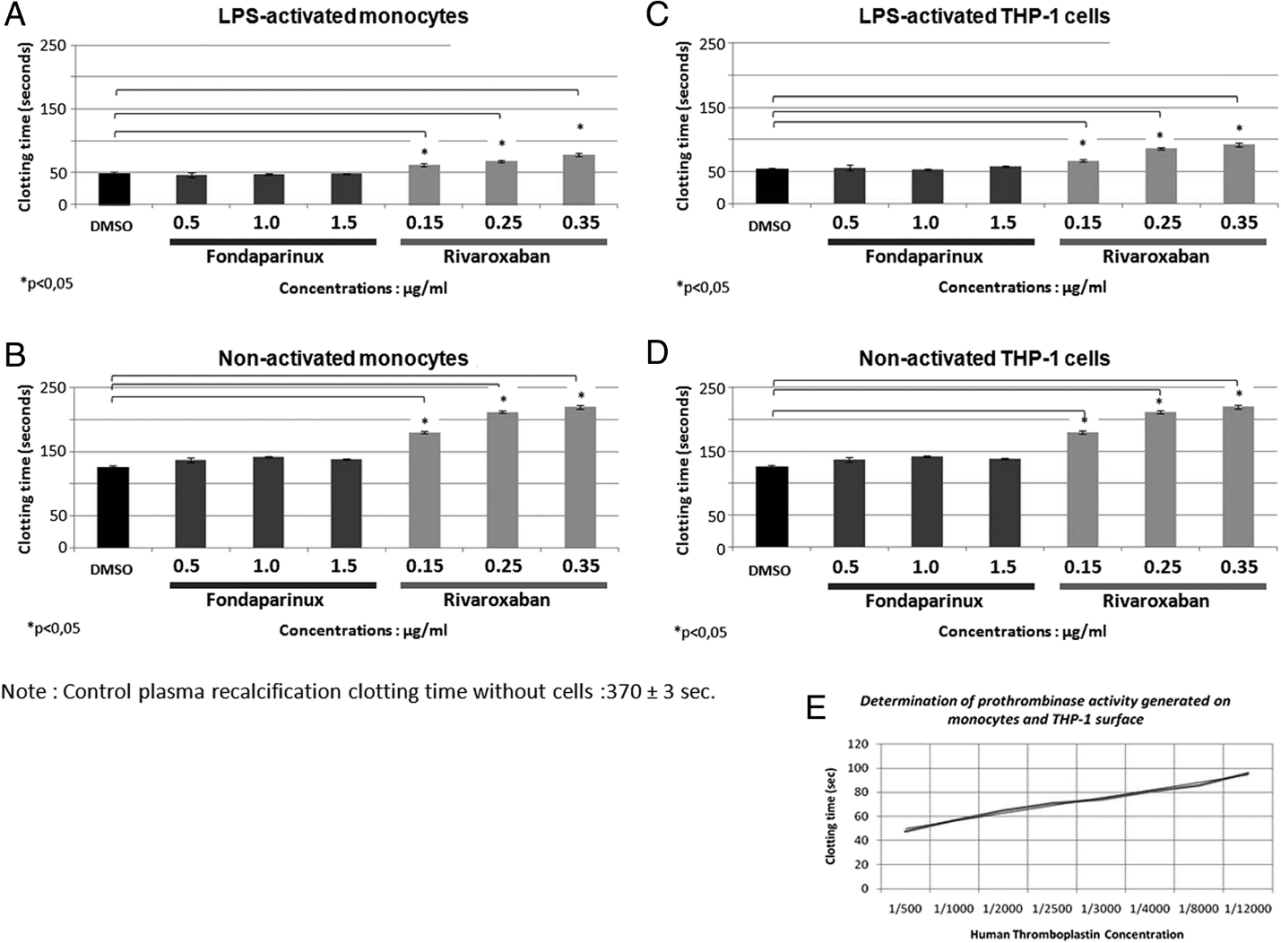
**Effect of rivaroxaban and fondaparinux on the cells triggering the plasma recalcification clotting time.** Non-activated or activated monocytes **(A and B)** and HP-1 cells **(C and D)** were used in the experiments. The cells were mixed with defibrinated plasma for 30 min and washed. Then CaCl_2_ and the cells were mixed with normal plasma and clotting time was recorded. The test was done in 5 independent experiments. **E**: Human thromboplastin was used to establish a linear curve in the same conditions without cells. *p < 0.05.

As shown in Figure 2, the addition of monocytes or THP-1 to normal plasma triggered coagulation as measured with the recalcification clotting time. The addition of the monocytes or THP-1 cells previously activated by LPS, the recalcification time was shortened approximately to 50 seconds in comparison to the addition of non-activated cells (approximately 125 seconds). This result was consistent with an increase in TF expression level on activated cells.

Then we tested two anti-FXa drugs on the same test. We observed that rivaroxaban inhibited the cell associated procoagulant activity and prolonged clotting in both normal monocytes and THP-1 cells with or without LPS activation (Figure 2A,B,C,D). We converted clotting time to human thromboplastin concentration (Figure 2E) and calculated percent inhibition of procoagulant activity by the two drugs. The results showed a clear difference between the two drugs (Table 1). Rivaroxaban was able to reduce concentration-dependently to 12% of the procoagulant activity on LPS-activated monocytes and THP-1, whereas fondaparinux did not modify the procoagulant activity in the same test.

**Table 1:** **Inhibition of procoagulant activity with clotting time**

	Rivaroxaban (μg/ml)			Fondaparinux (μg/ml)		
	0.15	0.25	0.35	0.5	1.0	1.5
Activated monocytes	30 ± 3*	16 ± 4*	12 ± 2*	105 ± 4	100 ± 6	105 ± 4
Activated THP-1	42 ± 2*	24 ± 3*	15 ± 1*	103 ± 7	102 ± 7	105 ± 8

Percent inhibition by two drugs of procoagulant activity was measured with clotting time assay compared to controls without drugs (100%). Mean ± SEM from 5 independent experiments. 100% was the prothrombinase generated by thromboplastin diluted 1/500 added to normal plasma. *p<0.05.

### Rivaroxaban and fondaparinux blocked thrombin generation induced by THP-1 cells

**Figure 3:**
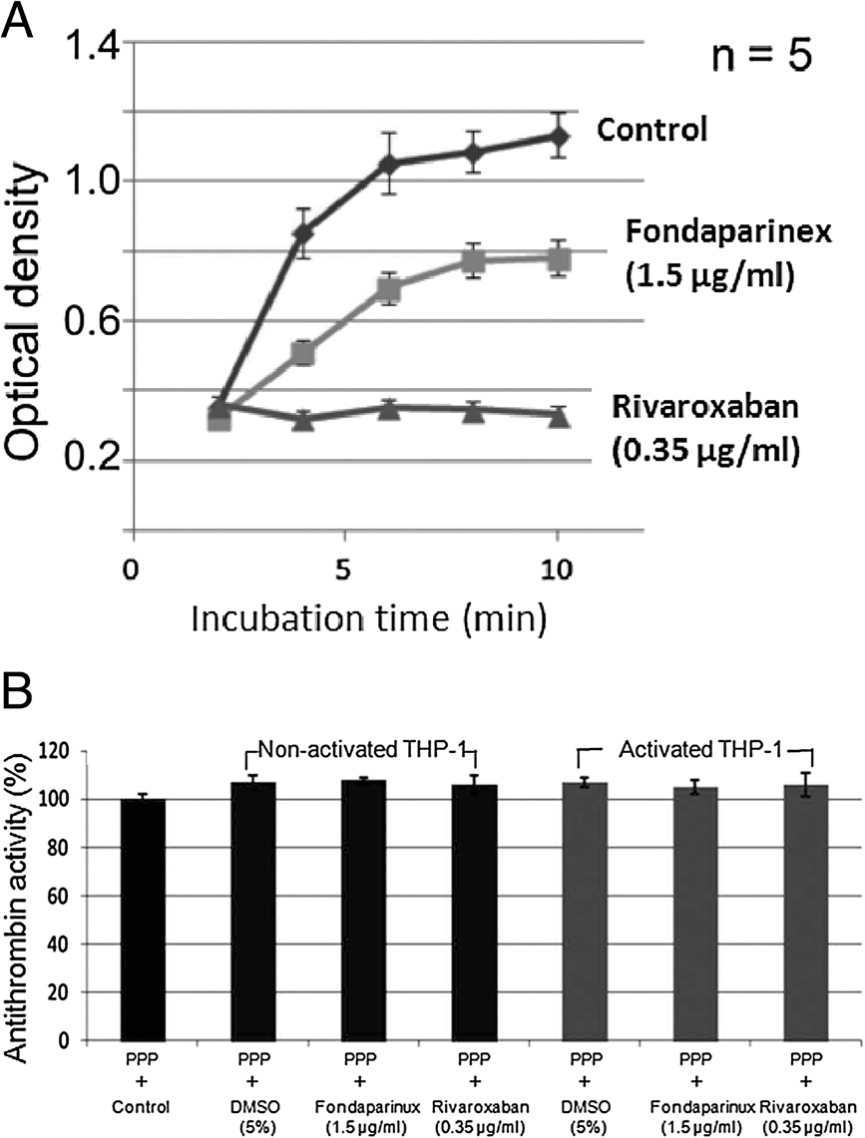
**Thrombin generation and AT III activity. (A)** Thrombin generation in platelet-poor plasma induced by the addition of LPS-activited THP-1. Time–dependent thrombin generation was evaluated using a synthetic substrate SQ 150. Activated THP-1 cells were washed and added to 240 μl of defibrinated plasma and CaCl_2_. Fondaprinux at 1.5 μg/ml or 0.35 μg/ml rivaroxaban were added into the test in order to evaluate their inhibitory effect. Optical density was measured. Significant differences were found among the three samples after 5 min reaction. **(B)** Antithrombin III activity in the absence or presence of rivaroxaban and fondaparinux in platelet-poor plasma (PPP) mixed with non-activated or PLS-activated THP-1 cells. The results are expressed as the percentage of AT activity in PPP. No statistical difference was found.

We further used thrombin substrate SQ 150 to evaluate thrombin generation after mixing the cells with defibrinogen plasma and CaCl_2_. As shown in Figure 3A, thrombin generation induced by the addition of LPS-stimulated THP-1 to plasma in the presence of calcium, is partially blocked in the presence of fondaparinux, while it is almost completely blocked in the presence of rivaroxaban.To exclude other influences such as the degradation of antithrombin III by secreted proteases from THP-1 cells, antithrombin levels were controlled using a STA Stachrom AT III kit. The addition of 0.35 μg/ml rivaroxaban or 1.5 μg/ml fondaparinux to activated or non-activated THP1 in defibrinated plasma did not modify the level of antithrombin III, excluding the suspected degradation of AT III protein in the test system (Figure 3B).

### Modulation of THP-1 cells-released cytokines by rivaroxaban and fondaparinux

**Figure 4:**
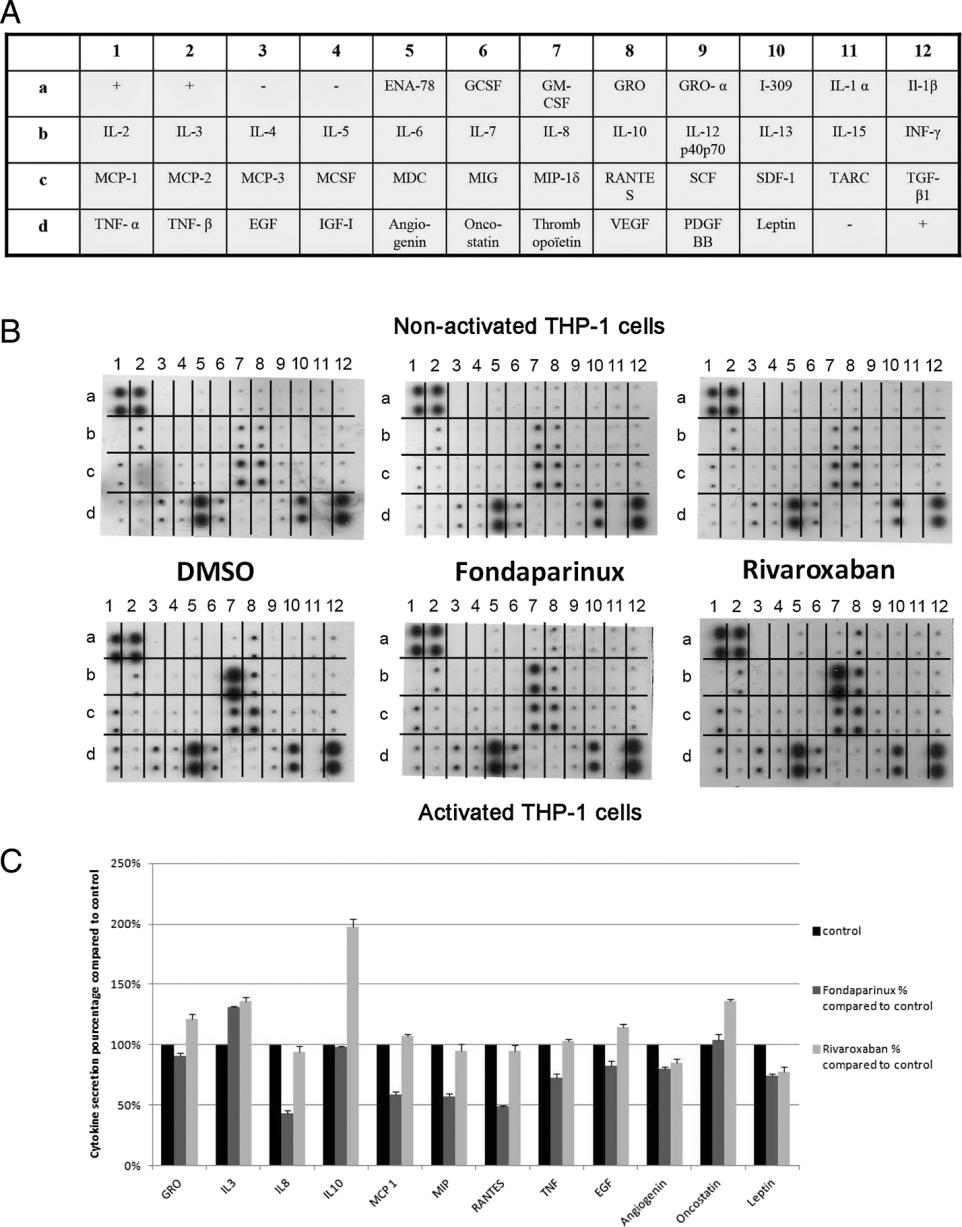
**Modulation of the cytokine secretion in THP-1 cells in response to fondaparinux and rivaroxaban. A**: Cytokine Array. **B**: Data of the cytokine assay. Non-activated and LPS-activated THP-1 cells were incubated in defibrinated plasma with PBS (control), 0.15 μg/ml fondaparinux or 0.35 μg/ml rivaroxaban. After 24 h, supernatants were assayed for cytokine production. All spots are in duplicate. a1, a2, and d12: positive controls. a3, a4 and d11: negative controls. **C**: the quantification by Image J of cytokines secretion by LPS-activated THP-1 cells. Two independent experiments were performed.

**Figure 5:**
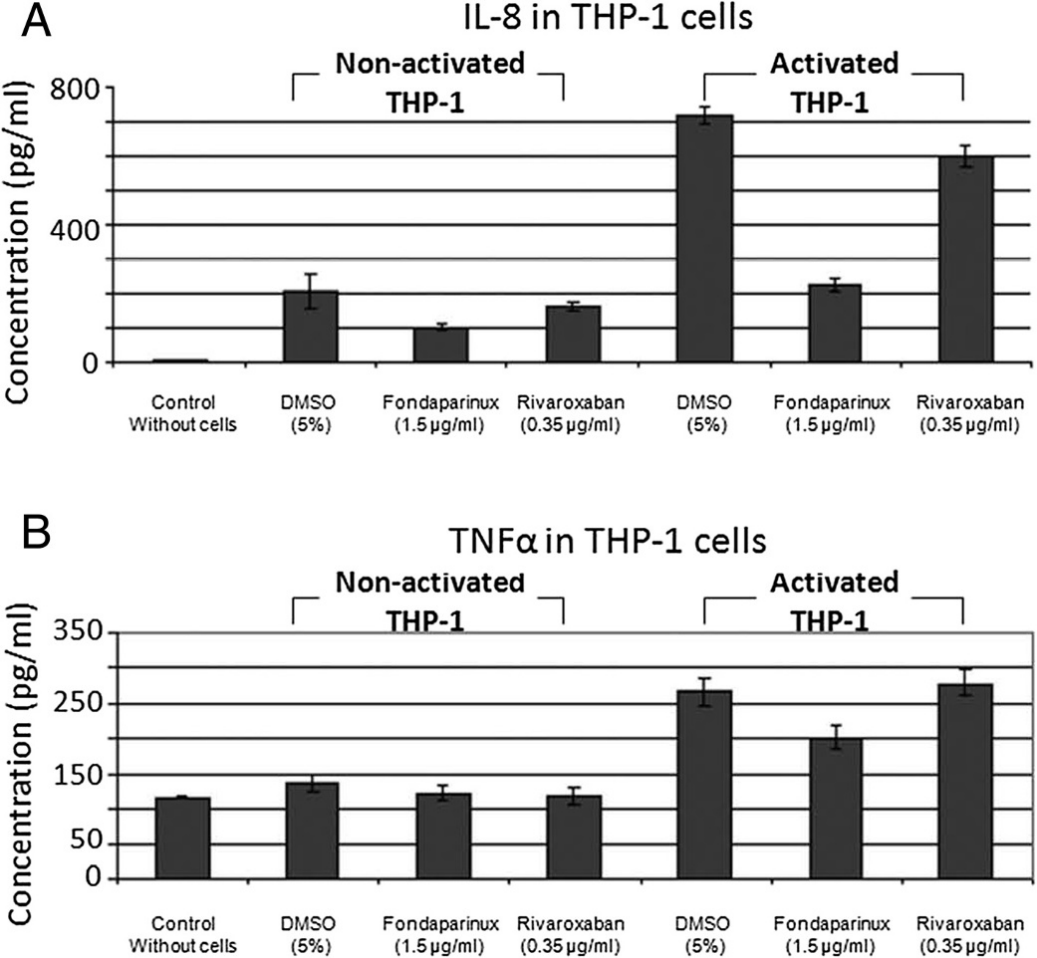
**ELISA quantification of IL-8 and TNFα in THP-1 cells.** Non-activated or LPS-activated THP-1 cells were incubated in defibrinated plasma in the presence of 0.35 μg/ml rivaroxaban or 1.5 μg/ml fondaparinux. After 24 hours, IL-8 in the supernatants was measured by ELISA. The data present is means ± SEM of 3 independent experiments. **A**: ELISA for IL-8. Statistic significance (p < 0.05) was found for the differences between non-activated cells versus control plasma; between non-activated versus LPS-activated cells; and between the activated cells treated by fondaparinux versus LPS-activated cells alone. **B**: ELISA for TNFα. Statistic significance (p < 0.05) was found for the differences between activated cells versus the control plasma; between activated cells treated by fondaparinux versus LPS-activated cells alone.

We explored the cytokine release by THP-1 cells during the generation of thrombin in defibrinated plasma and the inhibitory effect of the two drugs on cytokine secretion. The results showed that the activation of THP-1 cells by LPS induced a significant increase in IL-8, a moderate increase in Il-10, TNFα and Gro, and a slight increase in MIP-1α, Rantes, EGF and MCP-1 (Figure 4A,B).Both rivaroxaban and fondaparinux modified these changes in chemokine release. However, there are differences between the action of rivaroxaban and fondaparinux. Among these cytokines, we evaluated IL-8 and TNFα by ELISA and the results showed these differences. Fondaparinux was responsible for a decrease in IL-8 secretion by LPS activated THP-1 (43% by cytokine array and 36% by ELISA), whereas rivaroxaban had no effect on IL-8 secretion (94% by cytokine array and 84% by ELISA) (Figure 5A). Fondaparinux also induced a slight decrease in MCP-1, MIP, Rantes and TNFα secretion whereas rivaroxaban had no effect on this cytokine secretion. The action on TNFα secretion was also confirmed by ELISA (Figure 5B).

In addition, leptin and angiogenin secretion were moderately reduced by both rivaroxaban and fondaparinux (reduction < 30%). Rivaroxaban induced a strong increase in the anti-inflammatory cytokine IL-10 (198% of the secretion of activated THP-1 cells).

## Discussion

The results of flow cytometry showed that TF expression level on monocytes was not directly modified by the presence of rivaroxaban or fondaparinux. Then we evaluated enzymatic activity of FXa. In our study, the incubation with defibrinated plasma provides plasma coagulation factors to generate prothombinase activity on activated monocytes or THP-1 cell surface. The defibrination was carried out by reptilase and the procedure used in such way does not alter coagulation factors involved in thrombin generation and could avoid cell entrapment into fibrin network and cytokine adsorption into fibrin clot [35]. The results showed that LPS-activation upregulated TF expression in monocytes as well as their cell membrane-associated procoagulant activity. The test of prothrombinase activity bound to activated monocytes/macrophages using thrombin substrate SQ 150 demonstrated that the control LPS-activated THP-1 cells incubated with platelet-poor plasma induce a time dependent thrombin generation. Thrombin generation was totally blocked by rivaroxaban, whereas it was only partially inhibited by fondaparinux. The experiments with recalcification clotting time showed the same results that rivaroxaban significantly prolonged the clotting time showing its high efficiency in blocking the procoagulant activity of activated monocytes and THP-1 macrophages. In contrast, fondaparinux failed to significantly prolong the clotting time showing a much lower efficiency in blocking cell-associated procoagulant activity. The difference between fondaparinux and rivaroxaban effects on the inhibition of cell-associated procoagulant activity could be explained by different accessibility of these two drugs to FXa that binds to cell membranes. As a small chemical molecule rivaroxaban can easily get in contact with membrane-bound FXa and inhibit its activity, whereas the complex fondaparinux-AT with a heavy size is hard to get in contact with cell-associated factor Xa due to a problem of steric-hindrance. Our results are in agreement with the results of Kakar et al., showing that by inhibiting both free and bound factor Xa complexes, rivaroxaban prolongs activated partial thromboplastin time (aPTT) and prothrombin times (PT) in a concentration-dependent manner without antithrombin [36]. This differs from indirect FXa inhibitors, which only marginally prolonged aPTT and PT. The possible degradation of antithrombin by THP-1-releases proteases was excluded by AT III assay in agreement with Wiesel et al. [37]. The higher efficiency of rivaroxaban demonstrated in our study is interesting because clinical studies also reported the reduction of thrombotic events in the rivaroxaban treated patients group for hip and knee replacement in comparison to the enoxaparin-treated groups [38–41].

We evaluated the cytokine release in these cells since coagulation activation is known to stimulate the release of pro-inflammatory cytokines [42–44]. Coagulation proteases including FVIIa, FXa and thrombin bind and activate PARs which are present on endothelial and mononuclear cells, platelets, fibroblasts, and smooth muscle cells. We found that activation of THP-1 cells by LPS induced an important increase in IL-8, a moderate increase in Il-10, TNF and Gro, and a slight increase in MIP-1α, Rantes, EGF and MCP-1. Most of these cytokines are involved in angiogenesis. IL-8 regulats angiogenesis by enhancing endothelial cell survival, proliferation, and matrix metalloproteinase (MMPs) production [45]. TNFα is also a potent inducer of angiogenesis [46]. The upregulation of these cytokines and proangiogenic factors stimulate vascularization, monocyte accumulation and the progression of atherosclerotic plaques [47–49]. Importantly, vascularization in atherosclerotic plaque destabilizes the plaques and promotes its rupture, which causes thrombosis and acute ischemic events [50]. Histochemical examination of atherosclerotic plaque from autopsy specimens demonstrated a strong correlation between the severity of stenosis and plaque microvascular density [51]. Rantes was reported to participate in plaque destabilization by inducing the secretion of MMPs [52]. TNFα and Gro have been shown to be involved in atherosclerotic plaqueprogression [53]. Groα was among the top 10 most differentially expressed transcripts in peripheral blood mononuclear cells from patients with coronary artery disease in comparison with healthy controls. The Groα expression in macrophages within symptomatic carotid plaques was shown to enhance the release of matrix metalloproteinases which causes plaque embrittlement and angiogenesis [54].

The effects of rivaroxaban and fondaparinux on the cytokine release in activated macrophages are different. Rivaroxaban, only induced a strong increase in the anti-inflammatory cytokine IL-10 (198% of the secretion of activated THP-1 cells). This increase could be involved in the protection against atherosclerosis as suggested [55]. The effect of fondaparinux was fairly different. Fondaparinux had no effect on Il-10 production. However, it induced a clear decrease in IL-8 and to a less extent in Rantes and TNFα. These changes could be beneficial in clinic for reducing atherosclerotic lesions because IL-8 plays an important role in initiation and maintenance of the inflammatory microenvironment in atherosclerotic plaques [56]. Moreover, a slight inhibition of angiogenin transcription was observed when the cells had been treated by fondaparinux or rivaroxaban.

## Conclusion

Improving the efficiency of antithrombotic drugs is beneficial not only for cardiovascular diseases, but also for malignant diseases. Our data demonstrate that rivaroxaban, but not fondaparinux, inhibits efficiently the activation of thrombin by cell-associated procoagulant activity relevant to TF-FXa complex. This difference is most likely due to the steric hindrance that prevents the contact of the complex of fondaparinux-antithrombin with cell-associated FXa. This mechanistic difference argues for a higher efficacy of rivaroxaban as an antithrombotic drug in clinic.

## Materials and methods

### Plasma collection and inhibitor concentrations

Blood samples were collected from healthy volunteers in trisodium citrate (0.105 M) at ratio of 9:1 (volume/volume) and then centrifuged twice for 10 min (3000 g). Then plasmas were collected. Defibrination was performed as described by Dupuy et al. and Paysant et al. [57, 58]. Briefly, 3 ml of normal plasma was incubated with 100 μl reptilase (Stago, Asnières France) for 2 hours at room temperature. Then, the fibrin clot was agglomerated on a glass stick to obtain defibrinated plasma.

Rivaroxaban and fondaparinux were added to plasma at concentrations based on the Cmax observed in patients plasma treated by anti-Xa agent: i.e. 0.15, 0.25 and 0.35 μg/ml for rivaroxaban and 0.5, 1.0 and 1.5 μg/ml for fondaparinux.

### Macrophage culture and monocyte isolation

THP-1 macrophages (Human acute monocytic leukemia cell line) cells (ATCC) were cultured in RPMI 1640 culture medium containing 100 UI/ml penicillin (Eurobio), 100 μg/ml streptomycin (Eurobio), 2 mM L-glutamine (Eurobio) and 10% heat inactivated fetal calf serum (FCS, Eurobio). All cells were incubated in a humidified incubator at 95% air, 5% carbon dioxide, at 37°C and used within 20 passages.

Human mononuclear cells were isolated on a Ficoll gradient as previously described [59]. Briefly, blood from healthy volunteers was collected into polypropylene sterile tubes (Costar, Cambridge, MA) containing 1/5 (volume/volume) ACD (38 mM citric acid, 74 mM citrate, 136 mM glucose). After centrifugation (100 g, 10 min at room temperature), platelet-rich plasma was removed to minimize platelet contamination. Platelet-depleted blood was placed into 50 ml Leucosep tubes (Costar) that contained 15 ml of Ficoll (Eurobio). After centrifugation at 160 g for 10 min at room temperature, mononuclear cells were collected and washed three times in PBS (Eurobio). Cells were incubated for 1 hour at 37°C in 5% CO_2_, 95% water-saturated air mixture in 4 ml of AIM-V® medium (Gibco). Non-adherent cells (lymphocytes are the major type) were removed by two washes with PBS. Adherent cells (approximately 85-90% are monocytes) were then washed twice with PBS, detached with Accutase® cell detachment solution (eBiosciences), washed and adjusted at 1 × 10^6^/ml in PBS. Cells were counted by a Coulter counter.

### Monocyte and macrophage activation by lipopolysaccharide (LPS)

Ten μg of LPS (Sigma) were added to 1 ml of monocytes or THP-1 suspension (1 × 10^6^ cells/ml) and then incubated for 2 hours at 37°C with 5% CO_2_. Then cells were washed.

### Surface expression of TF on monocytes by immuno fluorescence

Briefly, monocytes activated or not with LPS, were incubated with defibrinated plasma in absence or presence of rivaroxaban or fondaparinux and were resuspended by pipetting. Then cells were washed twice in cold PBS. Approximately 1 × 10^6^ cells were incubated for 15 minutes at 4°C with 10 μl of FITC conjugated Mab anti-Human tissue factor (4508CJ: 100 μg/ml Ig) obtained from American Diagnostica. After two washes in PBS, the cell suspension was analyzed in a flow cytometer. The monocyte population was identified by gating the CD14 positive cells. A total of 10^4^ monocytes were analyzed. Results are expressed as a mean of fluorescence intensity (MFI). For each sample the background MFI produced by an isotype control antibody (eBioscience), was subtracted from the MFI value generated by the specific antibody.

### Prothrombinase activity of monocytes and macrophages treated or not by LPS

LPS-activated or non-activated monocytes or THP-1 were incubated with defibrinated plasma with either rivaroxaban or fondaparinux or saline (as control). Defibrinated plasma provides plasma coagulation factors to generate prothombinase activity on LPS-activated monocytes or THP-1 cell surface. Prothrombinase activity associated cells was evaluated by a 2 stage technique: 1) prothrombinase complex assembly on cell surface was performed by incubating 0.1 ml of a suspension of 1 × 10^6^ monocytes or THP-1 for 30 min at 37°C, treated or not with LPS, with 0.1 ml defibrinated plasma as a source of plasma coagulation factors. Defibrinated plasma was used in absence of anti-Xa agents (control) or in presence of rivaroxaban (used at the 3 indicated concentrations) or fondaparinux (used at the 3 indicated concentrations). Defibrination was required to avoid monocyte/macrophage sequestration in the fibrin clot and cytokine adsorption into fibrin clot. The defibrination was carried out by reptilase under conditions described by Hemker et al. [35]. This method does not alter coagulation factors involved in thrombin generation. Then cells were isolated by centrifugation, washed and suspended into 0.1 ml PBS. 2) Cells associated prothrombinase activity was evaluated by adding to a 0.1 ml cell suspension, 0.1 ml of plasma and 0.1 ml of CaCl_2_ 0.025 M. Results are expressed as a percentage of procoagulant activity. The prothrombinase generated by thromboplastin diluted 1/500 added to normal plasma was considered 100%.

### Thrombin generation evaluated on synthetic substrate by incubation of plasma and CaCl_2_ with LPS activated monocytes or THP-1

The 5 assays were carried out according to a slightly modified technique of Peyrou et al. [60]. Briefly, 60 μl of cells (1 × 10^6^ cells/ml) and 160 μl of CaCl_2_ 0.025 M were added to 240 μl of defibrinated platelet-poor plasma containing either 0.35 μg/ml of rivaroxaban or 1.5 μg/ml of fondaparinux or a corresponding solvent (control). Then, 75 μl of each sample were obtained every 2 minutes for 10 min and transferred into a pre-warmed (37°C) disposable tube containing 300 μl of 6 mM SQ 150 (Thrombin substrate, Stago®) in Tris buffer pH 8.4. After 3 minutes, the reaction was stopped by adding 15 μl of PPACK 1 mM. The supernatants optical density was evaluated at 405 nm with a Multiskan Ascent microplate reader from Labsystems (Brumath®, France). The lag time, the maximal thrombin concentration, and the time to maximal thrombin concentration were measured.

In order to evaluate all thrombin generated, without taking into account thrombin bound to fibrin clot, the experiments were performed using fibrinogen-depleted plasma.

### Determination of antithrombin (AT) in the supernatants of activated THP-1 incubated with defibrinated plasma

AT was determined using STA Stachrom AT III kit (Stago®). This test was carried out in order to investigate if proteases released by activated THP-1 could degrade AT, leading to a loss of its antithrombin activity.

### Measurement by cytokine array of inflammatory cytokines secreted by THP-1

Cytokine secretion was evaluated in a 24 well tissue culture plate Primaria Falcon (Becton Dickison): 300 μl of a suspension of 1 × 10^6^/ml THP1, either non activated or activated by lipopolysaccharide (LPS) (under conditions indicated above) were incubated in 300 μl defibrinated plasma in order to provide plasma coagulation factors in presence of 300 μl PBS (control), fondaparinux or rivaroxaban at 1.5 μg/ml and 0.35 μg/ml respectively. Incubation was done for 24 hours at 37°C, 5% CO_2_. Then media were collected and centrifuged at 1000 g for 10 minutes to discard cells.

Human Cytokine Antibody Array III kit (RayBiotech) was used. Forty-two different cytokines were evaluated: Epithelial Neutrophil-Activating peptide-78 (ENA-78), Granulocyte Colony-Stimulating Factor (GCSF), Granulocyte-Macrophage Colony Stimulating Factor (GM-CSF), Growth related oncoprotein (Gro), Groα, I-309, IL-1α, IL-1β, IL-2, IL-3, IL-4, IL-5, IL-6, IL-7, IL-8, IL-10, IL-12 p40p70, IL-13, IL-15, INFγ, Monocyte Chemotactic Protein-1 (MCP-1), MCP-2, MCP-3, Macrophage Colony Stimulating Factor (MCSF), Macrophage-Derived Chemokine (MDC), Monokine induced by IFNγ (MIG), Macrophage Inflammatory Protein-1α (MIP-1α), Regulated on Activation Normal T cell Expressed and Secreted (RANTES), human Stem Cell Factor (SCF), Stromal-Derived Factor-1 (SDF-1), Thymus and Activation- Regulated Chemokine (TARC), Transforming Growth Factor-β1 (TGF- β1), TNF-α, TNF-β, Epidermal Growth Factor (EGF), Insulin-like Growth Factor-I (IGF-I), Angiogenin, Oncostatin M, Thrombopoietin, Vascular Endothelial Growth Factor (VEGF), Platelet-Derived Growth Factor-BB (PDGF BB) and Leptin. Gro detected CXC Ligand 1 (CXCL1), CXCL2 and CXCL3; Groα detected only CXCL1. VEGF detected VEGF-165 and VEGF-121.

Briefly, 1 ml supernatant medium were incubated with arrayed antibody membranes, which were then exposed to specific biotin-antibody cocktail, following the manufacturer's instructions. Signals were detected using labeled-streptavidin by exposure on X-ray films.

We then compared the spot intensity on the samples: 1) activated or non-activated THP-1 cultured alone (to control basic cytokine secretion and cytokines provided by defibrinated plasma); 2) activated or non-activated THP-1 incubated with rivaroxaban at 0.35 μg/ml, 3) activated or non-activated THP-1 treated with fondaparinux at 1.5 μg/ml.

The area density of the spots was evaluated using image J (written in Java) which was downloaded from the National Center for Biotechnology Information. Signals were normalized against the positive controls of each membrane. The relative amount of each cytokine present in the cell culture medium is presented as the fold increase of the spot intensity in comparison to the supernatants of cytokines in control cells.

### Determination by ELISA of IL-8 and TNFα in the supernatants of non activated and LPS activated THP-1

IL-8 secretion level was measured by ELISA (kit Human CXCL8/IL-8 Quantikine® R&D Systems) in the supernatants of non-activated and activated THP-1 incubated with defibrinated plasma in conditions described above, in absence or presence of rivaroxaban at 0.35 μg/ml and 1.5 μg/ml fondaparinux. TNFα concentration in the supernatants was determined using Human TNFα ELISA Ready Set Go (eBioscience®) according to the manufacturer’s instructions.

### Statistical analysis

One-way ANOVA and Mann–Whitney U tests were used. The data are usually presented as mean ± SEM. The data of ELISA is presented as mean ± SD. A probability value of ≤0.05 was regarded as statistically significant.

## Abbreviations

LPS: Lipopolysaccharide
TF: Tissue factor
FX: Factor X
TFPI: Tissue factor pathway inhibitor
Mab: Monoclonal antibody
IL: Interleukin
TNF: Tumor necrosis factor
MMP: Matrix metalloproteinase
PPP: Platelet poor plasma.
